# Epidermal Growth Factor Receptor Inhibition Modulates the Microenvironment by Vascular Normalization to Improve Chemotherapy and Radiotherapy Efficacy

**DOI:** 10.1371/journal.pone.0006539

**Published:** 2009-08-06

**Authors:** George J. Cerniglia, Nabendu Pore, Jeff H. Tsai, Susan Schultz, Rosemarie Mick, Regine Choe, Xiaoman Xing, Turgut Durduran, Arjun G. Yodh, Sydney M. Evans, Cameron J. Koch, Stephen M. Hahn, Harry Quon, Chandra M. Sehgal, William M. F. Lee, Amit Maity

**Affiliations:** 1 Department of Radiation Oncology, University of Pennsylvania School of Medicine, Philadelphia, Pennsylvania, United States of America; 2 Medimmune LLC, Gaithersburg, Maryland, United States of America; 3 Department of Pharmacology, University of California San Diego, La Jolla, California, United States of America; 4 Department of Radiology, University of Pennsylvania School of Medicine, Philadelphia, Pennsylvania, United States of America; 5 Department of Biostatistics and Epidemiology, University of Pennsylvania School of Medicine, Philadelphia, Pennsylvania, United States of America; 6 Department of Physics and Astronomy, School of Arts and Sciences, University of Pennsylvania School of Medicine, Philadelphia, Pennsylvania, United States of America; 7 Department of Medicine, University of Pennsylvania School of Medicine, Philadelphia, Pennsylvania, United States of America; Dresden University of Technology, Germany

## Abstract

**Background:**

Epidermal growth factor receptor (EGFR) inhibitors have shown only modest clinical activity when used as single agents to treat cancers. They decrease tumor cell expression of hypoxia-inducible factor 1-α (HIF-1α) and vascular endothelial growth factor (VEGF). Hypothesizing that this might normalize tumor vasculature, we examined the effects of the EGFR inhibitor erlotinib on tumor vascular function, tumor microenvironment (TME) and chemotherapy and radiotherapy sensitivity.

**Methodology/Principal Findings:**

Erlotinib treatment of human tumor cells *in vitro* and mice bearing xenografts *in vivo* led to decreased HIF-1α and VEGF expression. Treatment altered xenograft vessel morphology assessed by confocal microscopy (following tomato lectin injection) and decreased vessel permeability (measured by Evan's blue extravasation), suggesting vascular normalization. Erlotinib increased tumor blood flow measured by Power Doppler ultrasound and decreased hypoxia measured by EF5 immunohistochemistry and tumor O_2_ saturation measured by optical spectroscopy. Predicting that these changes would improve drug delivery and increase response to chemotherapy and radiation, we performed tumor regrowth studies in nude mice with xenografts treated with erlotinib and either radiotherapy or the chemotherapeutic agent cisplatin. Erlotinib therapy followed by cisplatin led to synergistic inhibition of tumor growth compared with either treatment by itself (p<0.001). Treatment with erlotinib before cisplatin led to greater tumor growth inhibition than did treatment with cisplatin before erlotinib (p = 0.006). Erlotinib followed by radiation inhibited tumor regrowth to a greater degree than did radiation alone, although the interaction between erlotinib and radiation was not synergistic.

**Conclusions/Significance:**

EGFR inhibitors have shown clinical benefit when used in combination with conventional cytotoxic therapy. Our studies show that targeting tumor cells with EGFR inhibitors may modulate the TME via vascular normalization to increase response to chemotherapy and radiotherapy. These studies suggest ways to assess the response of tumors to EGFR inhibition using non-invasive imaging of the TME.

## Introduction

The idea of manipulating the tumor microenvironment (TME) to improve cancer therapy has been around for decades; however, finding ways in which to do this in the clinic has proven difficult. The response of tumors to radiation depends on factors in the TME including tumor cell-extracellular matrix interactions [1] and tumor oxygenation [2]. Efforts to decrease tumor hypoxia using hyperbaric oxygen have had limited success in increasing radiosensitivity [3]. In the 1970's, Folkman proposed the concept of targeting blood vessels within tumors to control their growth [4]. There are currently a number of anti-angiogenic drugs in clinical use but, used as single agents, these have had modest success in patient trials [5], [6]. More recently Jain and colleagues showed that anti-angiogenic therapy can result in a “normalization” of aberrant tumor vasculature in such as way as to improve oxygenation and blood flow that could enhance the efficacy of subsequent radiation and chemotherapy [7], [8]. Their approach relied on using agents that directly target vascular endothelial growth factor (VEGF) or its receptor (VEGFR) on endothelial cells. In the current study we use a different approach to alter the TME, to target the tumor cells to reduce VEGF secretion, thereby indirectly leading to vascular normalization.

The advent of molecularly targeted agents opens the possibility for inhibiting specific molecules and pathways critical for tumor growth, invasion and metastasis, and most of these agents target the tumor cells themselves. Tumor cells may be targeted by inhibiting the epidermal growth factor (EGFR). EGFR is overexpressed and activated in a variety of tumors and provides an attractive target for anti-cancer therapy (reviewed in [9]). In the early 1980's Mendelsohn and colleagues developed the monoclonal antibody C225 (now called cetuximab) and showed it to have efficacy in inhibiting cancer cell growth both *in vitro* and *in vivo* [10]. Since then, a variety of EGFR inhibitors, both monoclonal antibodies and small molecular kinase inhibitors such as gefitinib and erlotinib have been developed and tested in clinical trials. There is a clear connection between EGFR signaling and VEGF expression. EGF induces VEGF in many cell lines through increased VEGF mRNA transcription [11]–[13]. EGFR stimulation activates many downstream signaling pathways including the PI3K/Akt pathway [9]. Activated Akt increases expression of a key transcription factor, hypoxia-inducible factor-1α (HIF-1α) [14], [15]. One of the many transcriptional targets of HIF-1α is the VEGF gene. Conversely, pharmacological inhibition of EGFR can decrease VEGF expression and consequently angiogenesis in many tumor types [16]–[20].

Because EGFR inhibition can downregulate HIF-1α expression in tumor cells and decrease VEGF secretion, we hypothesized that erlotinib treatment would indirectly lead to vascular normalization and decrease tumor hypoxia. We explored the effects of erlotinib on the TME, specifically on vessel morphology, vascular permeability, tumor blood flow and tumor oxygenation. We found profound alterations in all of these parameters, which led us to investigate the effects of this agent on the subsequent response of tumors to chemotherapy and radiotherapy. Our results offer insight into how targeting tumor cells with EGFR inhibitors may modulate the TME to improve sensitivity to chemotherapy or radiotherapy. They have clinical implications about the sequencing and timing of these agents with conventional therapy and suggest ways to monitor their effects on the TME in the clinic using non-invasive imaging.

## Methods

### Ethics statement

All animal work was conducted according to relevant national and international guidelines. For details please refer to subsection entitled ***Mouse studies*** *.*

### Tissue Culture and Reagents

SQ20B head and neck squamous cell carcinoma, H226 and H292 non-small cell lung cancer cells were cultured in Dulbecco's modified Eagle's medium (DMEM, 4500 mg/liter glucose, Life Technologies, Inc.) containing 10% fetal bovine serum (Atlanta Biologicals) and grown in an incubator containing 5% carbon dioxide and 21% oxygen.

Erlotinib (Tarceva; OSI Pharmaceuticals; Melville, NY) was prepared as a 10 mM stock solution by taking an erlotinib tablet, crushing it and dissolving in DMSO. Cetuximab (Erbitux; ImClone, Branchburg, NJ) was prepared as directed by the manufacturer (2 mg/ml stock solution). siRNA was purchased from Dharmacon: EGFR siRNA SMARTpool (M-003114-03) and non-targeting #1-5 (control) siRNA (D-001210-02-20). Optimem media and Oligofectamine, purchased from LifeTechnologies, (Rockville, MD), were used as per the manufacturer's instructions.

### Protein Extraction, Western Blot Analysis

For protein isolation, cells were washed once with cold PBS containing 1 mM EDTA, then solubilized by adding lysis buffer (1% Triton X-100, 20 mM Tris, pH 7.6, 150 mM NaCl, 2 mM EDTA, 10% glycerol, 1 mM DTT, 1 mM orthovanadate, 2 mM PMSF) directly on the cells. Lysates were transferred into 1.5 ml Eppendorf tubes and centrifuged at 12,000 rpm for 10 minutes at 4°C. Supernatants were transferred to a fresh tube and frozen on dry ice. Protein concentrations were determined using a BCA Protein Assay kit (Pierce, Rockford, IL). For Western blotting, an equal amount of total protein was separated by SDS/PAGE on a 6% polyacrylamide gel.

For Western blotting, equal amounts of total protein were run in each lane of an SDS-PAGE gel (12% acrylamide). Each protein sample was mixed with an equal volume of 2× Laemmli buffer and boiled at 95°C for 5 minutes before loading onto the gel. After completion of gel electrophoresis, protein was transferred to a Hybond nitrocellulose membrane over one hour using a blotting apparatus. The following antibodies were used: monoclonal anti-phospho Akt antibody that recognizes P-S473 (New England Biolabs, Ipswich, MA), anti-Akt antibody, anti-HIFα antibody (clone H1α67, Novus Biologicals, Littleton, CO) at a dilution of 1∶1000, anti-β-actin antibody (Sigma, St. Louis, MO) at a 1∶1,000 dilution. The secondary antibody used for these blots was a goat anti-mouse antibody (Biorad, Hercules, CA). Antibody binding was detected by chemiluminescence using an ECL kit (Amersham Pharmacia, Piscataway, NJ).

### Mouse studies

Pathogen free female Ncr-nu/nu mice were obtained from Charles River Laboratory Inc. (Wilmington, MA) and housed in the vivarium of the University of Pennsylvania Laboratory Animal Resources. All the experiments were carried out in accordance with the protocols approved by University Institutional Animal Care and Use Committee guidelines. No more than 4 adult mice were housed in a cage to avoid overcrowding. The weight of the mice was monitored. As per IACUC regulations, a veterinarian would have to be contacted when any of the following occurred: weight dropped below 10% of the baseline, the mice appeared less active, depressed, sunken eyes, abnormal posture, mice had reduced feed or water intake (even lower than the restricted amounts), or showed signs of pain or distress. Mice were allowed to move freely most of the time. They were restrained only for brief periods, usually minutes, for specific research procedures. Appropriate anesthesia was administered to mice undergoing procedures that caused more than momentary or slight pain or distress. For tumor regrowth studies, the size of the subcutaneous tumors was monitored 3 times a week. Mice were sacrificed when tumors exceeded 10% of body weight.

To implant xenografts, mice at the age of five - seven weeks were subcutaneously injected with 1–2×10^6^ cells subcutaneously into the flank. Palpable tumors were generally observed 7–10 days after injection.

Prior to the start of experiments, mice were housed individually and daily food consumption was measured for 5 days. During the course of erlotinib treatment mice were also housed separately so that they could be fed a fixed amount of transgenic dough diet (Bioserve; Cat# S3472) daily. Erlotinib was mixed into the dough diet so that based on the average daily intake of each mouse; the targeted daily erlotinib dose was 50 mg/kg/day. During the treatment phase of the study, all the feed was eaten daily by each animal. No additional food was given. Body weight was measured at the start and end of feeding to ensure mice did not lose weight.

Cisplatin (Bedford Laboratories, Bedford, OH) was administered intraperitoneally. 1 ml of cisplatin stock (1 mg/ml) was diluted up in 10 ml of 0.9% saline. 250 µl was injected per mouse (average of 21 gm. per mouse). Bevacizumab (Avastin; Genentech, South San Francisco, CA) at 25 mg/ml and cisplatin were administered intraperitoneally.

### VEGF ELISA

Tumors were excised and weighed, then homogenized in 1 ml of 1x PBS (0.1% heparin). VEGF protein concentration was determined from tissue homogenate (100 µl) using a commercial kit (R & D Systems) according to the manufacturer's protocol. VEGF protein levels were normalized to their weights and plotted on y-axis.

### Vascular permeability assay

Evans blue (30 mg/kg) was injected intravenously and allowed to circulate for 6 hrs. Thereafter mice were sacrificed and tumors were excised, dried (60°C, 16 hr), and weighed before Evans blue extraction in 1 ml of formamide at 55°C for 16 hr. Evans blue content was quantified by spectrophotometer reading at 620 nm.

### Power Doppler studies

Contrast-enhanced power Doppler imaging was performed as described previously [21]. In brief, mice bearing SQ20B xenografts were anesthetized and injected with 0.02 ml of microbubble contrast agent (Definity, Lantheus Medical Imaging, Billerica, MA, USA) and power Doppler imaging was performed using a broadband 7–15 MHz probe (HDI500 SonoCT, Philips, Bothell, WA, USA). Power Doppler images were acquired at a frame rate of 0.5 Hz to minimize microbubble destruction by the imaging ultrasound pulses. The Doppler signal from the inflowing contrast agent was visible in the images in color superimposed on the grayscale image of the tumor. The power Doppler images at peak enhancement were analyzed to determine **p**ercentage **a**rea of the tumor with **f**low (PAF) and **c**olor-**w**eighted **f**low **a**rea (CWFA), representing relative blood volume flowing through the unit volume of the tumor in the image plane [22], [23].

### In vitro and in vivo radiation

Cells in exponential growth phase were counted and plated in 60-mm dishes containing 4 mL medium. The cells were allowed to attach for 4 hours, then erlotinib was added to cultures one hour before radiation. Cells were irradiated with a Mark I cesium irradiator (J.L. Shepherd, San Fernando, CA) at a dose rate of 1.6 Gy/min. Colonies containing>50 cells were stained and counted 10 to 14 days after irradiation. The surviving fraction was calculated by dividing the number of colonies formed by the total number of cells plated, times plating efficiency. Each point on the survival curve represents the mean surviving fraction from at least three replicates.

Irradiation of the flank bearing the tumor was performed using a 250 kV orthovoltage irradiator (Philips RT 250) at a dose rate of 2.63 Gy/min through a 0.2-mm copper filter. The source-to-tumor target distance was 30 cm with adequate shielding of non-tumor sites.

### Broadband diffuse reflectance spectroscopy

To quantify tissue optical properties and determine tissue hemoglobin oxygen saturation (SO_2_), broadband diffuse reflectance spectrometric measurements were performed prior to the start of erlotinib therapy and after 4 days of therapy. The instrument and the analysis algorithm have been described in detail in previous publications [24], [25]. Briefly, the instrument consists of a 250-W quartz tungsten halogen lamp, a hand-held surface contact fiber optic probe, a spectrograph, and a liquid nitrogen–cooled CCD camera to image the reflectance spectra from multiple detection fibers simultaneously. For this experiment, the reflectance spectra in 600–800 nm range from source-detector distances between 1.2 to 4.0 mm were used for data analysis. The data was fit to the analytical solutions of photon diffusion equation for semi-infinite geometry to calculate tissue concentrations of oxy-hemoglobin (HbO2) and deoxy-hemoglobin (Hb). From this information, the total hemoglobin concentration (THC = cHbO2+cHb) and the tissue hemoglobin oxygen saturation (SO_2_ = cHbO_2_/THC) were derived. In previous study, the broadband diffuse reflectance spectroscopy was validated through reproducing the oxy-hemoglobin dissociation curve for a tissue phantom of mouse erythrocytes, and by correlating pO_2_ measured by Eppendorf pO_2_ histograph and SO_2_ of *in vivo* mice with varying oxygenation. The dissociation curve matched the published values closely (< 5% difference) and the correlation coefficient of *in vivo* mice was 0.90 (23).

### Detection of hypoxia with EF5

EF5 is a 2-nitroimidazole that forms covalent protein adducts in viable hypoxic cells in a manner that is inversely proportional to oxygen concentration in the physiologic range [26]. Details regarding its use in assessing tumor oxygenation in human tumors and human tumor xenografts in rodent models are provided elsewhere [27]–[29]. EF5 studies were performed after five days of erlotinib therapy.

Briefly, mice were injected with 10 mmol/L drug in 2.4% ethanol and 5% dextrose intravenously (0.01 ml/g body weight), followed by an equal volume intraperitoneal injection 30 minutes later. Three hours after the first EF5 injection, mice were euthanized. The tumor was resected and frozen in OTC compound (Sakura Finetek Torrance, CA) by using dry ice. For analysis of hypoxia, 10 µm sections were cut onto poly-L-lysine–coated slides, fixed in 4% paraformaldehyde for 1 hour, and then rinsed and blocked for 2 hours at room temperature. Slides were stained with Cy3-conjugated ELK3-51, a mouse monoclonal antibody to EF5. Just prior to imaging, tissue sections were dipped briefly into a 25 µM Hoechst 33342 solution. This stains the nuclei, which can then also be imaged, prior to the imaging of EF5, over the same coordinates.

### Determination of tumor growth delay

Xenografts were grown as described above. The mice were started on erlotinib, given cisplatin or irradiated when the tumors reached approximately 1 cm in diameter. Mice were examined twice weekly for evaluation of tumor growth. Tumors were measured with calipers in three mutually perpendicular diameters (a, b, and c) and the volume was calculated as V = (π/6) x a x b x c.

### Confocal microscopy and tomato lectin studies

Mice bearing xenografts tumors were fed with erlotinib diet (treated) or diet only (control) for 4 days. Then mice were intravenously injected FITC conjugated tomato (*Lycoperscion esculentum*) lectin (Vector Laboratories) to label perfused vessels. After 30 minutes, tumors were excised and frozen in OCT (Sakura Fineteck, Torrance, CA) using liquid nitrogen. Thereafter, 100 µm section(s) were used to perform confocal microscopy.

### Cisplatin determination in tissue samples

Samples were sent to ESA Laboratories. Inc. (Chelmsford, MA) for cisplatin determination using graphite furnace atomic absorption spectrometry [30]. The tissue samples were prepared using an acid digestion method in which the sample is heated in HNO_3_ and H_2_O_2_. The sample was then brought up to final volume in dilute HCl and analyzed by AAS Graphite Furnace (Perkin Elmer 600) with a detection limit of 25 ng. Prior to cisplatin determination on mice treated with the drug, standardization was performed using tumors taken from five non-cisplatin treated mice.

### Statistical analysis

Models were constructed to analyze the effects on the treatment on tumor growth rate, which was summarized as the time to reach a target tumor volume. If the data were complete (e.g., all animals reached the target volume) then a linear regression model was employed. However, if the data were censored (e.g., animals expired or were sacrificed prior to reaching the target volume) a Cox regression model was employed. As described previously [31], a regression model was fit to time to tumor volume data that included terms to target tumor volume data that included terms to estimate the individual (main) effects of each treatment and the interaction of these two treatments on the tumor regrowth. For the cisplatin/erlotinib experiments, the linear model took the form:
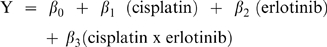



Y = days to reach a target tumor volume during the observation period, cisplatin, and erlotinib are indicators for the treatment received (1 = yes, 0 = no) and cisplatin x erlotinib is an interaction term. The test of interaction between cisplatin and erlotinib was conducted on the interaction term using a one-sided Wald statistic to determine whether β_3_>0, indicating synergy, defined as a more than additive effect. Due to censoring (i.e., animals expired or were sacrificed prior to reaching the target volume) of approximately 30% of animals in the erlotinib and radiation experiments, a Cox regression model was fit to time to target tumor volume data that included terms for the main effects of erlotinib and radiation and the interaction between these two treatments. Wald test of synergy was similar to that described above. With no censoring of animals in the cisplatin/erlotinib sequence experiments, the comparison of time to reach a target tumor volume among control, erlotinib→cisplatin and cisplatin → erlotinib groups was performed by ANOVA. Post-hoc paired group comparisons were performed by Scheffe testing to control the overall type I error rate. A significance level of 0.05 was considered to be statistically significant. Regression modeling and group comparisons, as described above, were performed in SPSS 12.0 (SPSS, Inc., Chicago, IL).

Unpaired Student's t-tests were used for comparisons between groups of tumors or dishes of cells. Paired t-tests were used for comparison within individual tumors before and after treatment. These statistics were calculated using KaleidaGraph (version 3.6.2; Synergy Software, Reading, PA).

## Results

### EGFR inhibition downregulates HIF-1α and VEGF expression in vitro and in vivo

**Figure 1:**
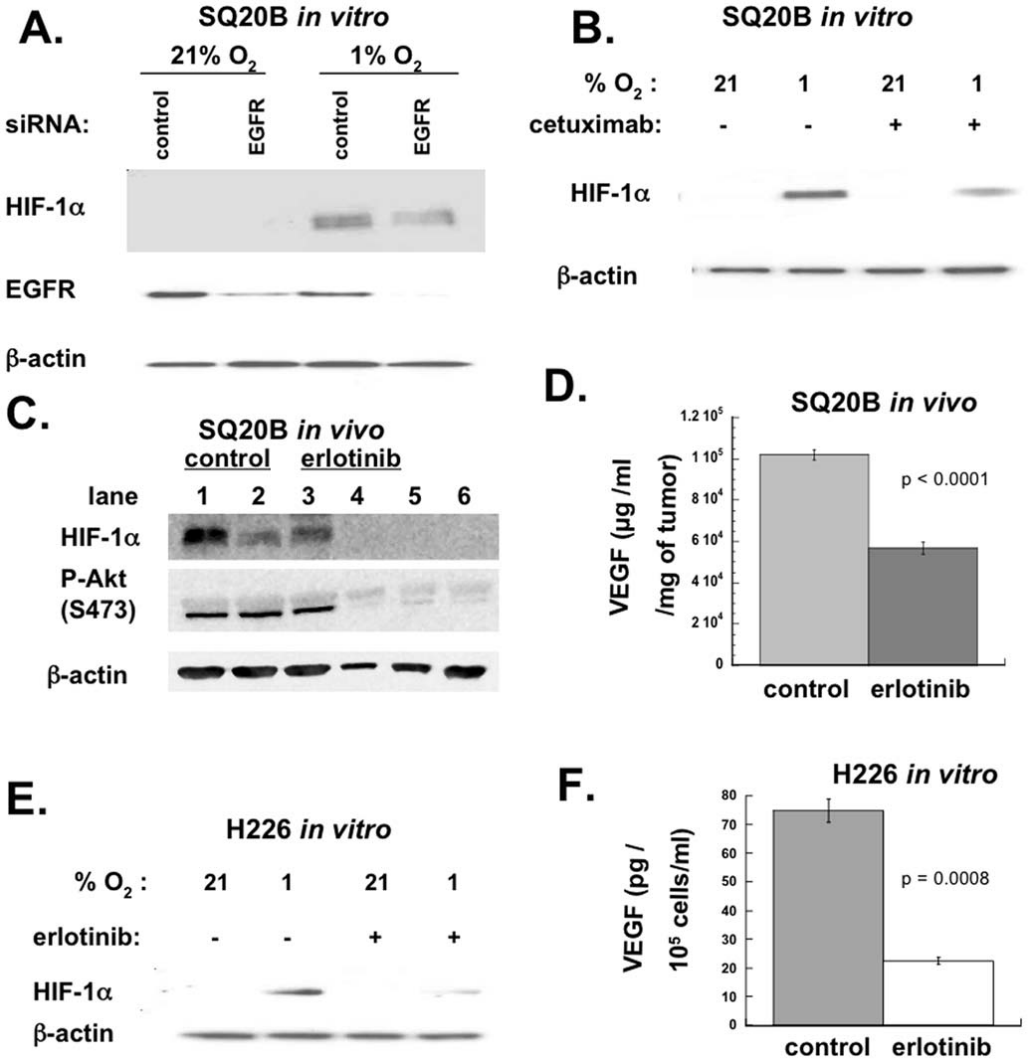
EGFR inhibition downregulates VEGF and HIF-1 *in vitro* and *in vivo*. SQ20B cells were seeded at 25% confluence and cultured overnight. The next morning, cells were transfected with 150 nanomoles siRNA (either EGFR SmartPool or control non-targeted) in Optimem transfection medium using Oligofectamine. After 24 hours, the transfection medium was replaced with fresh, prewarmed culture medium. 24 hours after this (48 hours after transfection), cells were exposed to either 21% or 1% oxygen. Three hours later they were harvested and Western blotting was performed. (B) SQ20B cells were treated with 10 nM cetuximab or DMSO (control) for 16 hrs, and then exposed to either 21% or 1% oxygen. Three hours later they were harvested and Western blotting was performed. (C) Nude mice were injected subcutaneously in the flank with SQ20B cells to form xenografts. When the tumors reached a size of 100–150 mm^3^ (approximately 7–10 days after injection), half the mice (lanes 4–6) were started on an erlotinib-containing diet (50 mg/kg/day). After 4 days of feeding, mice were sacrificed, and the tumors were removed. Half of each tumor was lysed in protein lysis buffer, and Western blotting was performed for pAkt (S473) and HIF-1α. Each lane represents a tumor from a different mouse. (D) The other half of each tumor from (E) was homogenized in PBS, and then ELISA for VEGF was performed. VEGF level was normalized to tumor weights. Data shown represent mean values from 3 control mice and 3 erlotinib-treated mice. Error bars represent standard error of the mean. p value was obtained using Student's t-test. (E) H226 cells were treated with 10 µM erlotinib or DMSO (control) for 16 hrs, and then exposed to either 21% or 1% oxygen. Three hours later they were harvested and Western blotting was performed. (F) H226 cells were seeded then later that day they were treated with erlotinib (10 µM) or DMSO (control). 24 hours later, the media was changed and replaced with media containing 1% serum with or without erlotinib. Cells were exposed to 1% oxygen. 16 hours later, aliquots of supernatant were removed from dishes, and ELISA for VEGF was performed. ELISA values were normalized to the number of cells present. Data shown represent mean values. Error bars represent standard error of the mean. p value was obtained using Student's t-test.

Our previous work demonstrated that small molecule EGFR inhibitors including erlotinib decreased VEGF mRNA expression, decreased secretion of VEGF protein, and blunted HIF-1α induction in response to hypoxia in SQ20B head and neck squamous cell carcinoma cells [20]. We extended these studies by using siRNA directed against EGFR. This also led to a decrease in HIF-1α induction in response to 1% oxygen by approximately 50% (Fig. 1A). Likewise, treatment of these cells with the anti-EGFR monoclonal antibody cetuximab also attenuated HIF-1α induction in response to hypoxia (Fig. 1B).

To determine the effects of EGFR inhibition *in vivo* and to examine their consequences, we implanted SQ20B cells subcutaneously in nude mice. When these cells formed tumors approximately 1 cm in diameter, host mice were fed a control diet or a diet containing erlotinib for 4 days to deliver 50 mg/kg/day. After five days, mice were sacrificed, and tumors were excised. Each tumor was divided in two and processed for either Western blotting or ELISA. Western blotting showed that erlotinib almost completely abolished p-Akt and HIF-1α expression *in vivo* (Fig 1C), similar to what was seen *in vitro*. Erlotinib also led to an approximately a 50% decrease in VEGF expression (Fig. 1D).

In order to show that these results were not unique to SQ20B cells, we repeated the experiments in two other cells lines. Figs. 1E and 1F show that erlotinib blunted both HIF-1α expression and secretion of VEGF protein in H226 lung carcinoma cells. Similar results were seen in H292 lung carcinoma cells (Fig. S1).

### Erlotinib alters vessel morphology and permeability in vivo

**Figure 2:**
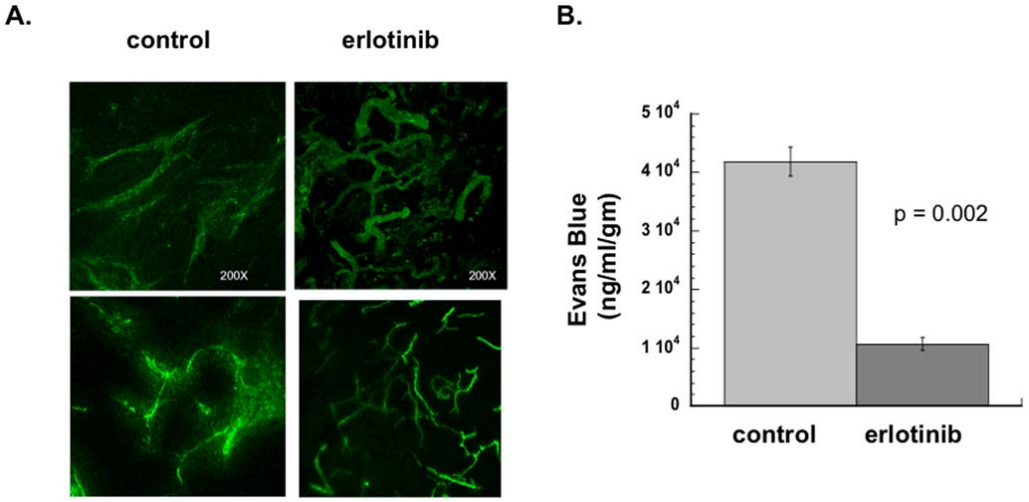
Erlotinib alters vascular morphology and increases vascular permeability *in vivo*. (A) Nude mice were injected subcutaneously in the flank with SQ20B cells to form xenografts. When tumors reached a size of ∼1 cm in diameter, some of the mice were started on an erlotinib-containing diet (50 mg/kg/day). After 4 days of erlotinib treatment (or control diet), FITC-conjugated tomato lectin was injected via tail vein. The mice were euthanized, and then a 100 µm thick section of each tumor was viewed under confocal microscope. The projected photomicrograph (200× total magnification) of sections of tumors from 2 different erlotinib-treated and 2 control mice are shown (B) Tumors were grown as described in (A). Evans blue dye was injected intravenously. Six hours later mice were sacrificed and tumors were excised. Dye was extracted, then quantified by reading at 620 nm in a spectrophotometer. Data shown represent mean of 3 control mice and 3 erlotinib-treated mice. Error bars represent standard error of the mean. p value was obtained using Student's t-test.

Because HIF-1α and VEGF are intimately associated with tumor vascularization, we examined the effect of erlotinib on tumor vessels. Mice bearing SQ20B xenografts were injected with tomato lectin prior to being sacrificed, and confocal microscopy was performed on the excised tumors. This showed a change in the vascular morphology (Fig. 2A) with the vessels in the erlotinib-treated tumors appearing more sharply and discretely outlined compare to vessels in control tumors. Control tumors showed more lectin staining surrounding the vessels. This suggested that erlotinib caused changes in vessel structure and/or permeability, resulting in less leakage of lectin into the interstitium. To assess this directly, we injected Evans blue dye and quantified the amount that extravasated into the tumor interstitium. We found that erlotinib treatment significantly decreased vascular permeability (Fig. 2B).

### Erlotinib increases tumor blood flow

**Figure 3:**
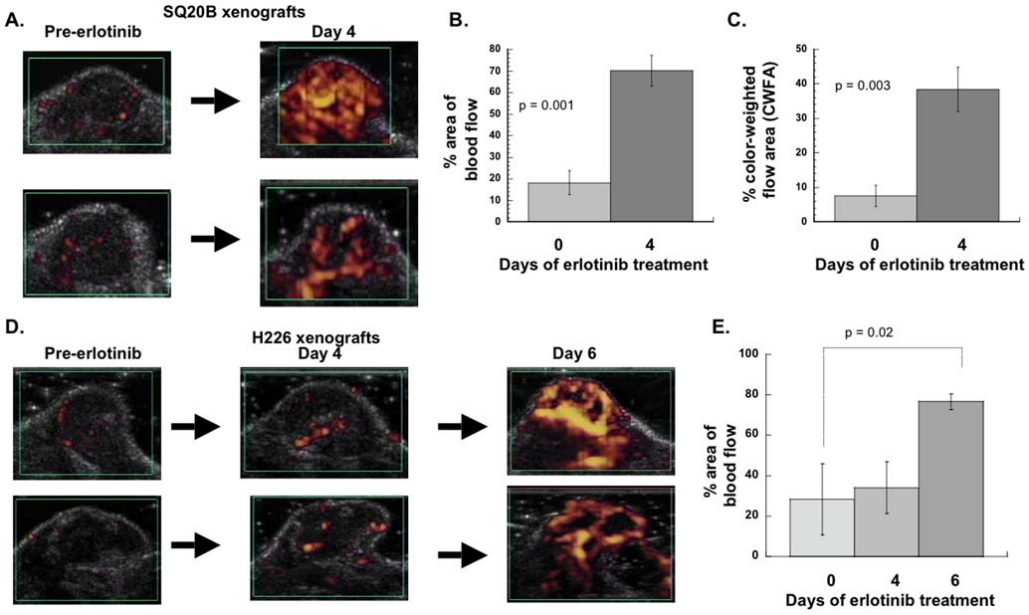
Erlotinib increases blood flow *in vivo*. Mice were injected subcutaneously in the flank with SQ20B cells (panels A–C) or H226 cell (panels D, E) to form xenografts. When tumors reached a size of ∼1 cm in diameter, mice were anesthetized and injected with microbubble contrast agent, then power Doppler ultrasound was performed. Mice were then started on an erlotinib-containing diet (50 mg/kg/day). Repeat Doppler measurements were performed at day 4 in all mice and also at day 6 for mice with H226 xenografts. (A) Doppler flow in two mice with SQ20B xenografts imaged pre-erlotinib and at day 4 of erlotinib treatment. Area of contrast enhancement (yellow pixels) signifies the perfused region of the TME. (B) Percentage area of flow (PAF) was quantified in 4 SQ20B xenografts. (C) Area of color-weighted flow area (CWFA) was quantified in same 4 xenografts used in (B). (D) Doppler flow in two mice with H226 xenografts imaged pre-erlotinib and at days 4 and 6 of erlotinib treatment. (E) PAF was quantified in 4 mice with H226 xenografts (including two shown in (D)). Figures in (B), (C) and (E) show the mean values before and after treatment with erlotinib; bars represent the standard error of the mean. p values were calculated using paired Student's t-test.

The data in Fig. 2 suggested that erlotinib decreased vascular permeability and changed vessel morphology. In order to determine what effect this might have on tumor blood flow, we used power Doppler ultrasound. By day 4 of erlotinib therapy, most tumors showed an increase in tumor blood flow (Fig. 3A). We quantified this effect using 2 different parameters, percentage **a**rea of the tumor with **f**low (PAF) and color-weighted flow area (CWFA), which are shown in Figs. 3B and 3C respectively. Using either of these parameters we saw a statistically significant increase in blood flow. In control mice not treated with erlotinib, there was no increase in tumor blood flow with time (data not shown).

We repeated the Doppler studies using a different xenograft model, H226 non-small cell lung cancer cells. In this model erlotinib treatment also led to an improvement in blood flow (Fig. 3D); however, in general this took longer than in SQ20B xenografts, on the order of 6 days rather than 4 days. Analysis of percentage area of tumor with flow confirmed a statistically significant increase in tumor blood flow from day 0 to day 6, but there was no difference between day 0 and day 4 (Fig. 3E). Analysis of CWFA also confirmed the increase in blood flow (data not shown).

### Bevacizumab increases tumor blood flow

Vessel normalization and the consequent increase in tumor vascular flow were originally seen using VEGFR2 antagonists to directly target tumor blood vessels [8]. To see whether improvements in tumor vascular function through targeting EGFR achieved comparable effects as anti-VEGF therapy in our model, we treated mice with SQ20B xenografts with the anti-VEFR monoclonal antibody bevacizumab. Treatment of this agent led to an increase in blood flow 4 days after injection, similar to that seen with erlotinib (**Fig. S2**).

### Erlotinib decreases hypoxia and increases SO_2_ in vivo

**Figure 4:**
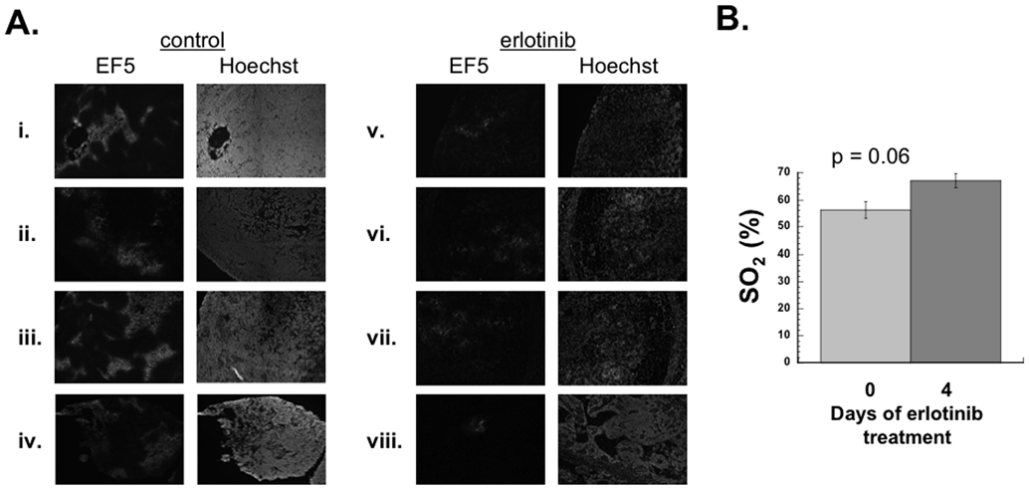
Erlotinib decreases hypoxia *in vivo*. (A) Eight mice were injected subcutaneously in the flank with SQ20B cells to form xenografts. Half the mice were started on erlotinib feed when the tumors reached a size of ∼1 cm in diameter; the other half were fed control diet. After 4 days, mice were injected with EF5, sacrificed 3 hours later and the tumors harvested. Tumors were stained with Hoechst 33342 and with the Cy3 conjugated anti-EF5 Elk3-51 antibody. Images represent representative sections of tumors taken from 4 erlotinib-treated mice (panels i-iv) and from 4 control mice (panels v-viii). (B) A different set of mice was injected subcutaneously with SQ20B cells to form xenografts. When the tumors reached ∼2 cm in diameter, Doppler ultrasound and broadband diffuse reflectance spectroscopy (to determine tissue hemoglobin oxygen saturation (SO_2_)) were performed. Mice were then started on erlotinib-containing diet. Four days later Doppler and optical measurements were repeated. Mean tissue hemoglobin oxygen saturation (SO_2_) for these 7 tumors pre- and post-erlotinib is shown. Bars represent the standard error of the mean. p values were calculated using paired Student's t-test.

As vascularization could affect tumor oxygenation, we measured hypoxia in our tumors using the 2-nitroimidazole, EF5. This agent binds to regions of hypoxia in a manner that is inversely proportional to oxygen concentration [26]. EF5 was injected after 5 days of erlotinib treatment, and then mice were sacrificed three hours afterwards. Images were acquired after immunohistochemical staining for EF5 (Fig. 4A). Tumors from 4 different control mice showed patchy regions of high EF5 binding, consistent with heterogeneous binding and the presence of markedly hypoxic tumor regions (panels i-iv). In mice treated with erlotinib the EF5 staining was noticeably less prominent, consistent with decreased hypoxia (Fig. 4A).

As an alternate method of assessing tumor oxygenation, we measured tissue hemoglobin oxygen saturation (SO_2_) in a group of mice prior to erlotinib therapy and on day 5 of erlotinib therapy. Tumors were allowed to grow to approximately 1 cm in diameter before erlotinib was started in order to have tumors of sufficient size to perform broadband diffuse reflectance spectroscopy before and after therapy. These tumors (n = 7) also underwent power Doppler ultrasound to assess tumor blood flow. The mean tumor blood flow increased after 4 days of erlotinib treatment (Fig. 4B). Optical spectroscopy showed the pre-erlotinib SO_2_ to be 56.4±3.1% (mean value±SEM). This increased to 67.1±2.7% (mean value±SEM) after 4 days of erlotinib therapy (Fig. 4C; p = 0.06; Student's t-test). Of the seven tumors in this experiment, six showed an increase in tumor vascular perfusion after 4 days of erlotinib therapy. All six of these tumors showed an increase in SO_2_. In the single tumor that showed a decrease in vascular perfusion, the SO_2_ also decreased. Therefore, there was a perfect correlation between increasing vascular perfusion and increasing SO_2_. These tissue hemoglobin oxygen saturation data support our hypothesis that erlotinib improves tumor oxygenation in tumors via improved vascular perfusion.

### Effect of erlotinib on response to cisplatin in SQ20B head and neck squamous cell xenografts

**Figure 5:**
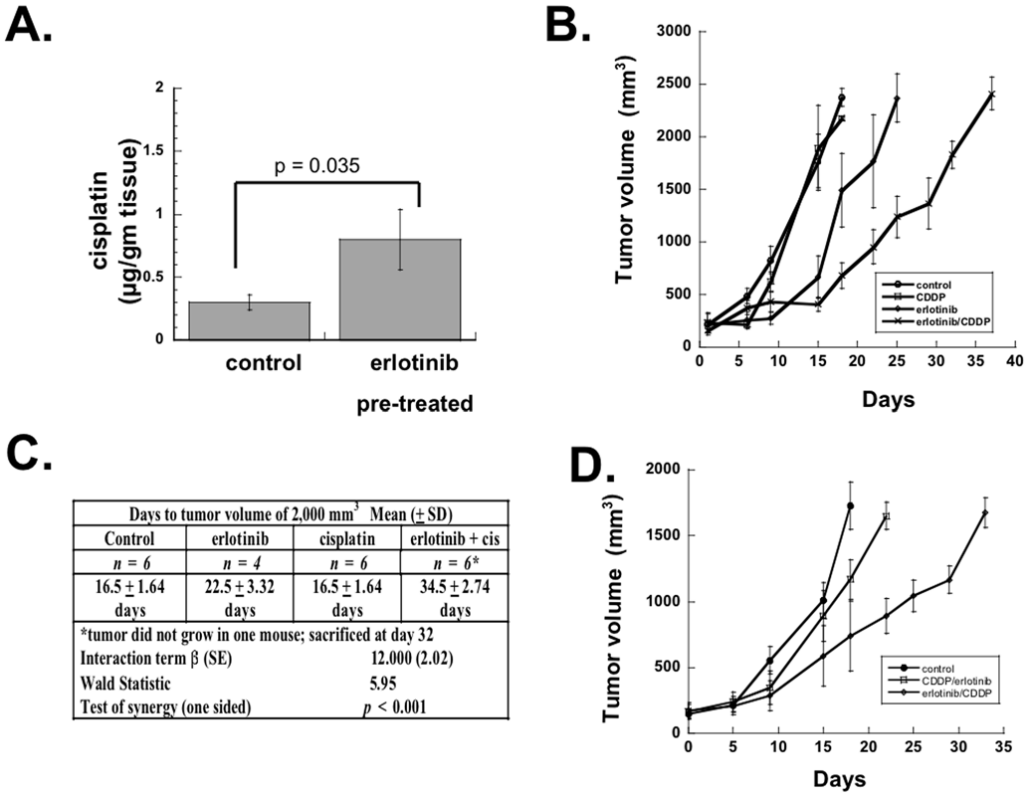
Effect of erlotinib with cisplatin on tumor regrowth of xenografts. (A) Mice were subcutaneously implanted with SQ20B cells. When tumors reached a size of ∼5 mm in diameter, therapy was started. One group of mice (five mice) received a single dose of cisplatin (CDDP) intraperitoneally (1 mg/kg). Three hours later, the mice were sacrificed, and tumors were removed and snap frozen in liquid nitrogen. The second group of mice was fed an erlotinib-containing diet, and then on day 5 the mice were injected with CDDP intraperitoneally (1 mg/kg). Three hours later, the mice were sacrificed, and tumors were removed and snap frozen in liquid nitrogen. (B) Mice were subcutaneously implanted with SQ20B cells. When tumors reached a size of ∼5 mm in diameter, therapy was started. One quarter of the mice received no therapy (control). One quarter received a single dose of cisplatin (CDDP) intraperitoneally (1 mg/kg) on Day 1. One quarter received erlotinib feed on days 1 through 4 and the morning of day 5 followed by a single dose of CDDP on day 5. One quarter received erlotinib feed starting on day 1 and continuing through the morning of day 5 but no CDDP. Erlotinib was not given from day 6 onward for any group. (C) Test of synergy for regrowth delay in time to reach tumor volume of 2,000 mm^3^ (D) Mice were subcutaneously implanted with SQ20B cells. When tumors reached ∼5 mm in diameter, a third of the mice were given a single injection of CDDP (on Day 1) then later that day started on an erlotinib-containing diet that continued through day 5. A third of the mice were given erlotinib feed on days 1 through 4 and the morning of day 5 followed by a single dose of CDDP on day 5. Erlotinib was not given from day 6 onward for any group. A third received no therapy (control). Mean tumor volume±standard deviation of time to reach 1,250 mm^3^ were 16.5±3.21, 17.1±4.22 and 26.0±6.40 days for control, CDDP ->erlotinib, erlotinib ->CDDP groups respectively. The three groups were significantly different (p = 0.0001; ANOVA) with the erlotinib ->CDDP group having a significantly longer time period as compared with both control (p = 0.003) and CDDP ->erlotinib (p = 0.006) groups.

A predicted consequence of increased blood flow to a tumor would be enhanced drug delivery, which would have obvious clinical implications for the combination of anti-EGFR therapy and chemotherapy. The agent cisplatin is the most commonly used drug in the treatment of head and neck squamous cell cancers; hence, we used this drug to test our hypothesis. SQ20B xenografts were grown in nude mice. When these reached a size of approximately 5 mm, the control set of mice were given a single dose of cisplatin. Three hours later they were sacrificed, and the tumors were removed. The experimental group of mice was started on erlotinib-containing feed, then after four days (on day 5), the mice were sacrificed and the tumors removed. Tumors were flash frozen in liquid nitrogen and sent to an outside laboratory to determine the level of cisplatin in the tumors using graphite furnace atomic absorption spectrophotometry. Fig. 5A shows that the mice that were pre-treated with erlotinib had a higher level of cisplatin in their tumors than did the control mice.

With a higher level of cisplatin within the tumors, we predicted that this would lead to increased efficacy in tumor control. In order to determine whether this was the case, we gave mice with SQ20B xenografts a single dose of cisplatin or a 4-day course of erlotinib followed by a dose of cisplatin on day 5. Fig. 5B shows that the dose of cisplatin that we used was subtherapeutic, resulting in no change in tumor regrowth compared with control mice. Erlotinib by itself had some effect on retarding tumor regrowth, but the single dose of cisplatin administered after 4 days of erlotinib therapy had a much greater effect. The effect between erlotinib and cisplatin was more than additive (test of synergy; p<0.001 by linear regression) as shown in Fig. 5C.

If the synergistic effect of erlotinib combined with cisplatin was primarily due to changes in the TME induced by erlotinib leading to increased cisplatin cytotoxicity, then the order in which these two agents are given should be critical in determining outcome. To test this, we repeated the tumor regrowth assay, this time giving one set of mice erlotinib for 4 days followed by a dose of cisplatin on day 5 and another set the same dose of cisplatin followed by 4 days of erlotinib. The results, shown in Fig. 5D, unequivocally demonstrate that the sequencing is critical. Erlotinib followed by cisplatin had a greater effect on inhibiting tumor regrowth than the reverse combination. There was a statistically significant difference among the three groups in the time taken for tumors to reach a volume of 1,250 mm^3^ (p = 0.001) with a significantly longer time period for erlotinib→cisplatin as compared to both control (p = 0.003; ANOVA) and cisplatin→erlotinib (p = 0.006) groups.

### Effects of erlotinib on radiation response

**Figure 6:**
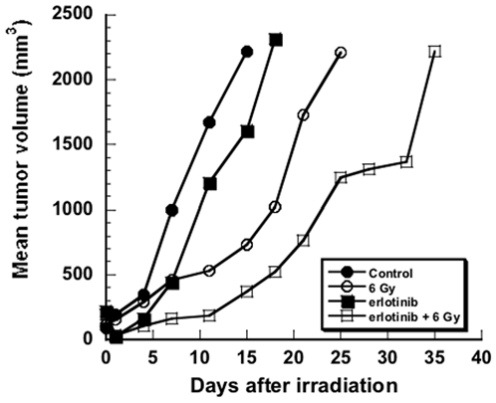
Effect of erlotinib on *in vivo* and *in vitro* radiation response. Mice were implanted with SQ20B xenografts. When tumors reached a size of ∼1 cm in diameter, half the mice were started on erlotinib feed; half were not. After 4 days of this feed, half the erlotinib–treated and half the control mice received 6 Gy; half of each group received no radiation. Mean values for tumor size are plotted on the graph.

Improvement in hypoxia should theoretically lead to improved radiation response *in vivo*, an idea that we tested using the tumor regrowth delay assay. Mice bearing SQ20B tumors were assigned to one of 4 treatment groups (radiation plus erlotinib, radiation alone, erlotinib alone, or mock treatment). Mice were pretreated for 4 days with erlotinib prior to irradiation (6 Gy). The results are shown in Fig. 6A. Due to censoring of animals, survival analysis was employed to evaluate treatment interaction. The median time to reach a tumor volume of 1,000 mm^3^ was 17 days in both the control group and the erlotinib group. The median time increased to 24 days in the radiation and to 34 days in the radiation+erlotinib group. The effects on tumor regrowth with the combination therapy appeared to be at most additive, as the test for synergy was not statistically significant (p = 0.24, Cox regression analysis). We also investigated the effect of erlotinib on *in vitro* radiosensitivity. Using a standard clonogenic survival assay, we found that erlotinib had no effect on the radiation survival curve in SQ20B cells (Fig. S3).

## Discussion

The concept of vascular normalization, as proposed by Jain [7], has relied primarily on the idea of targeting the tumor vasculature via anti-VEGF or VEGFR agents. According to this model, tumors often express high levels of VEGF, leading to aberrant vasculature that provide inadequate blood flow to tumors. A decrease in VEGF activity, for example by using the anti-VEGFR agent AZD2171 can reduce interstitial fluid pressure and “normalize” blood vessel morphology, leading to improved vascular perfusion [7], [8]. In this report we investigate the idea of using agents targeting the tumor cells themselves. We chose the EGFR inhibitor erlotinib, but other agents that indirectly decrease VEGF expression may show the same effect. Data from many laboratories indicate that inhibition of EGFR signaling can decrease expression of HIF-1α and VEGF [16]–[20], most likely through the PI3K pathway. We confirmed that erlotinib inhibited HIF-1α and VEGF both *in vitro* and *in vivo* in SQ20B squamous cell carcinoma cells. The decrease in VEGF expression *in vivo* was accompanied by a change in vessel morphology (Fig. 2A), a decrease in vascular permeability (Fig. 2B) and an increase in tumor blood flow (Fig. 3). Therefore, we hypothesize that erlotinib treatment has an indirect effect on the vessels resulting from a decrease in VEGF secretion by the tumor cells. Further evidence supporting the idea that VEGF is the downstream effector responsible for the improved blood flow comes from the fact that in the same SQ20B tumor model, the anti-VEGF antibody bevacizumab led to a similar improvement in blood flow (Fig. S1).

We investigated the consequences of improved tumor blood flow in response to erlotinib. One prediction is that erlotinib pre-treatment would alter the TME, allowing for better delivery of chemotherapy (e.g. cisplatin) and improved therapeutic effect. Our data confirmed both. Erlotinib pre-treatment did increase cisplatin levels in the tumors (Fig. 5A). A single dose of cisplatin that by itself had no effect on tumor regrowth was strongly synergistic with erlotinib in inhibiting tumor regrowth. Furthermore, the timing of cisplatin relative to erlotinib was critical. Erlotinib followed by cisplatin had a much greater effect on inhibition of tumor regrowth than did the reverse combination. This result is consistent with the idea that erlotinib alters the TME in a way that allows cisplatin to have a greater effect.

We also found that erlotinib treatment decreased hypoxia as it improved blood flow. We demonstrated this by using both EF5 immunohistochemistry and by measuring tissue hemoglobin saturation. Solomon *et al*. [32] had previously shown that the EGFR inhibitor gefitinib decreased intratumoral hypoxia in A431 xenografts using ^18^F-FAZA PET scanning with the nitroimidazole FAZA. Another group showed that gefitinib improved tumor oxygenation in ErbB2 expressing breast cancer xenografts using EF5 flow cytometry [33]. Our results are entirely consistent with these studies. Furthermore, we offer a mechanism for this phenomenon, namely that EGFR inhibition downregulates VEGF expression, leading to vascular normalization and improved blood flow.

A predicted consequence of decreased hypoxia would be improved response to radiation therapy. Krause *et al.* provided evidence that increased oxygenation after targeting of the EGFR by C225 might contribute to the improvement in local control following radiation [34]. Indeed we found that there was a greater effect on tumor regrowth when erlotinib was followed by 6 Gy of radiation than with either treatment by itself, although this was not a synergistic effect. We hypothesize that this lack of synergy may be a result of the heterogeneity of tumor response. In contrast to the effects *in vivo*, erlotinib had no effect *in vitro* in radiosensitizing SQ20B cells (Fig. S3). We acknowledge that other groups have found that EGFR inhibition can increase *in vitro* radiosensitivity. Cetuximab has also been shown to augment radiation-induced cell killing and to decrease the proportion of cells in S phase, a more radioresistant phase [35]. Several groups have shown that EGFR inhibition can decrease DNA damage repair, perhaps by altering an interaction between EGFR and DNA-PK [17], [36], [37]. Chinnayan *et al.* showed that erlotinib radiosensitized cells *in vitro*, an effect that might have occurred through a combination of increased apoptosis, cell cycle arrest, and changes in DNA damage repair [38]. Notwithstanding these results, our findings suggest that an additional mechanism may also be operative in the improved radiation response seen *in vivo* with EGFR inhibition.

Clinical studies using EGFR inhibitors as monotherapy have been disappointing. Approximately 10% of patients with non-small cell lung cancer (NSCLC) (those harboring an activating mutation of EGFR) show a dramatic shrinkage in response to the inhibitor gefitinib [39], [40], but this is unlikely to lead to long-term cure. Most clinical trials (e.g. IDEAL, SWOG0023) have failed to show a survival advantage with EGFR inhibitors as monotherapy in NSCLC (reviewed in [9]).

EGFR inhibitors have also been tested in combination with chemotherapy. Numerous preclinical studies have shown that EGFR inhibitors can sensitize cells to chemotherapy [41]–[45] and to radiotherapy [17], [38], [46], [47]. However, several trials (e.g. TRIBUTE, TALENT, INTACT) have failed to show a benefit to small molecule EGFR inhibitors in combination with chemotherapy in NSCLC (reviewed in [48]). A phase III trial in patients with advanced pancreatic carcinoma showed that adding erlotinib to gemcitabine resulted in a very small improvement in overall survival (on the order of weeks) compared with gemcitabine alone [49]. In one phase III trial, the addition of cetuximab to cisplatin in metastatic/recurrent head and neck squamous cell carcinoma improved tumor response rate but did not impact survival [50]. However, a phase III trial in patients with platinum-resistant recurrent or metastatic head and neck squamous cell carcinoma found that the addition of cetuximab to platinum-based chemotherapy significantly prolonged median overall survival (7.3 months to 10.1 months) [51]. One can only speculate as to why there are such disparate results in these trials. In part, it may relate to redundant signal transduction pathways that occur in cancers in patients that experimental models may not reflect. Mechanisms of resistance in specific tumors could involve activation of insulin-like growth factor (IGF)-1 signaling, activation of Met signaling, loss of PTEN, or mutation of K-Ras that can compensate for the therapeutic inhibition of EGFR signaling. Additionally, certain tumor types may be more susceptible to signaling modulation by EGFR inhibition, perhaps because they are more reliant on the EGFR pathway for survival. Head and neck tumors have a high rate of EGFR overexpression [52], which may suggest that these tumors as a class are more EGFR-dependent than other tumor types. Also, it has been suggested that the method by which EGFR is inhibited (antibody versus small molecule inhibitor) may be important in what downstream events are inhibited.

The only randomized phase III trial published to date examining radiation versus radiation plus an EGFR inhibitor used cetuximab in locally advanced head and neck squamous cell carcinoma [53]. This trial showed an improvement in local control and survival favoring the combined modality group. As discussed above, EGFR inhibition has been shown to have numerous effects that could lead to increased in vitro radiosensitization. However, this does not preclude an effect on the TME. It is conceivable that the scheduling of weekly cetuximab on this trial produced sufficient vascular normalization to improve oxygenation and increase radiation response.

Our findings may have clinical implications. Improvement in blood flow leading to better drug delivery or increased tumor oxygenation offers a rationale as to why these EGFR inhibitors might perhaps be started prior to cytotoxic therapy and continued through this therapy. Our pre-clinical findings suggest the idea of imaging the TME prior to and after 5–7 days of EGFR inhibitor therapy in order to assess whether there has been any modulation of the TME and whether in patients in whom this occurs there is any improvement in outcome. Non-invasive imaging techniques are currently available in the clinic that could be used to assess tumor vascularity (i.e. DCE MRI, Power Doppler) and/or oxygenation (e.g. PET scanning with hypoxia sensitive tracers) [54]–[57]; hence, our hypothesis could be tested in a pilot clinical trial.

## Supporting Information

Figure S1 (5.82 MB TIF)

Figure S2 (5.82 MB TIF)

Figure S3 (5.82 MB TIF)
