# Supplementary figures and images for: Epidermal Growth Factor Receptor Inhibition Modulates the Microenvironment by Vascular Normalization to Improve Chemotherapy and Radiotherapy Efficacy
Source: PLoS One. 2009 Aug 6;4(8):e6539. doi: 10.1371/journal.pone.0006539 (PMC2716529; doi:10.1371/journal.pone.0006539)

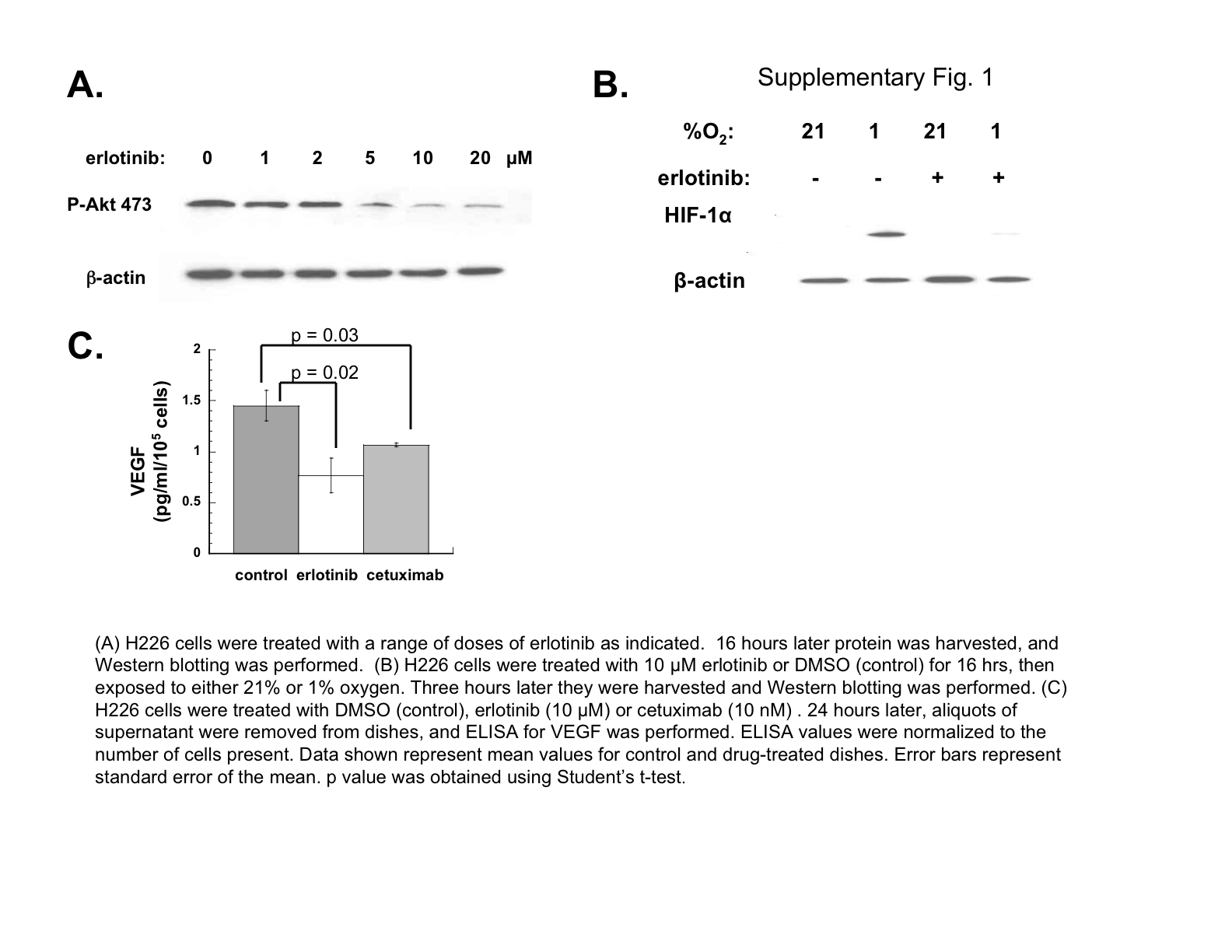

Supplement: Figure S1 — (5.82 MB TIF) [file pone.0006539.s001.tif]

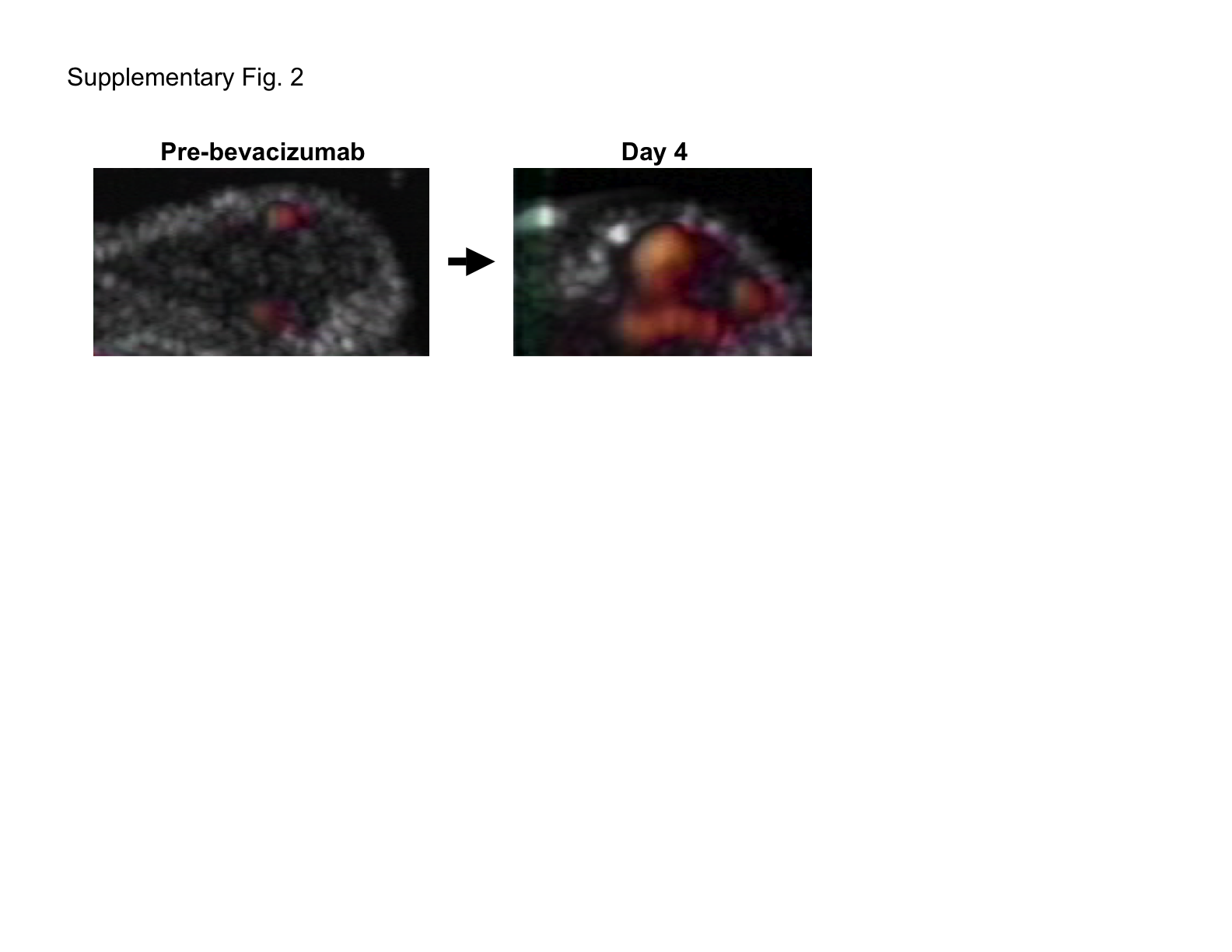

Supplement: Figure S2 — (5.82 MB TIF) [file pone.0006539.s002.tif]

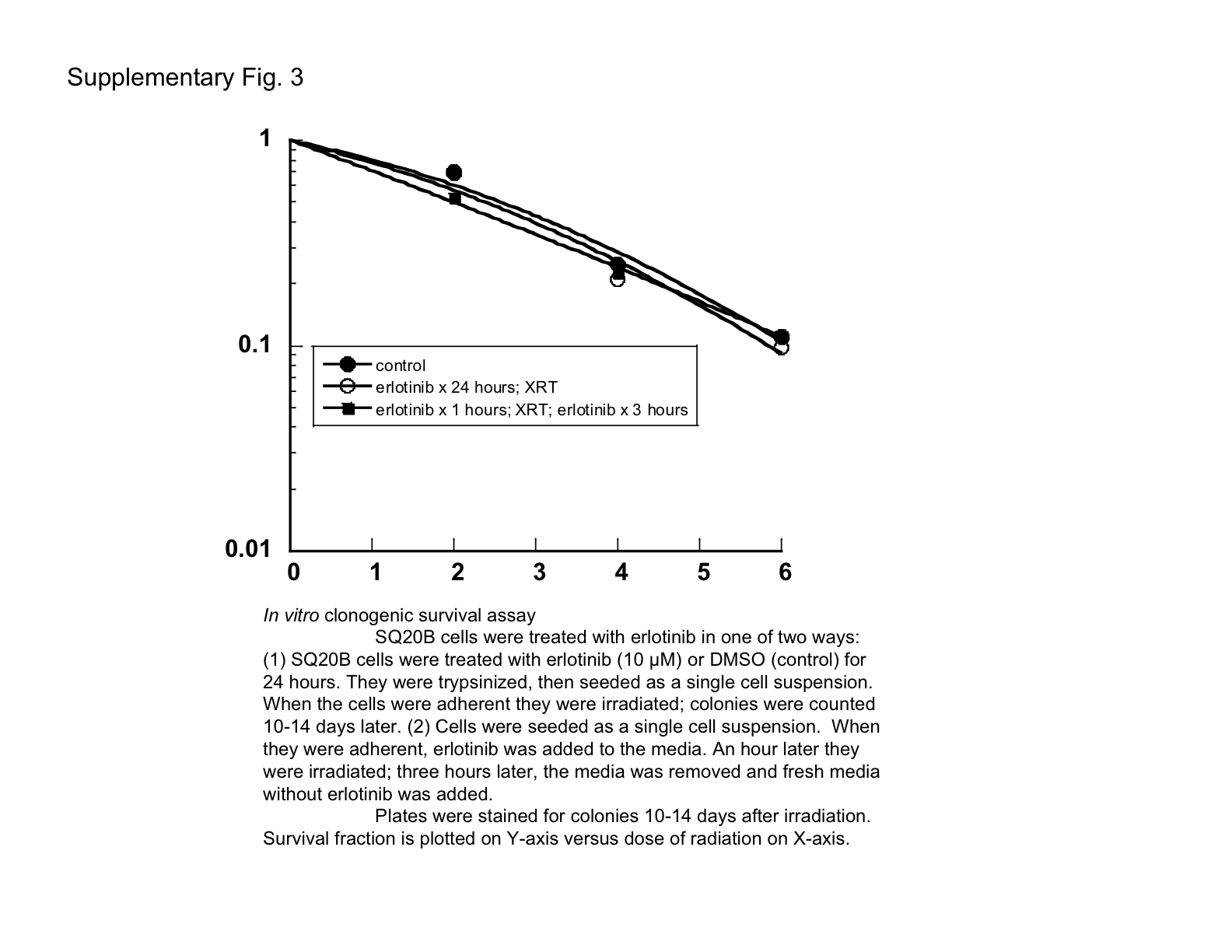

Supplement: Figure S3 — (5.82 MB TIF) [file pone.0006539.s003.tif]
